# Mr. Crowfoot, in Reply to Drs. Mossman & Langslow

**Published:** 1802-04

**Authors:** W. Crowfoot

**Affiliations:** Beccles


### *To the Editors of the Medical and Physical Journal.*

GENTLEMEN,

I HOPE you will indulge me with inserting in the Medical Journal, a few Remarks upon Dr. Moffman's *unmerited censure*, and some very plain Observations upon Dr. Langslow's Reply; with which I propose to take my leave of him as a Disputant, perfectly convinced in my own mind, that Controversy is impracticable and Refutation useless. I presume Dr. M. to many of your readers, may seem to betray a want of that candour, the absence of which he so feelingly laments in others; otherwise, it is hardly possible he should, in so unqualified a manner, so seriously and severely blame a man he knows nothing of; and with the most unwarrantable freedom declare, that "whatever my reasons are for *not consulting with Dr. Langslow*, the disclosure cannot do honour to my judgment or my feelings." If, as Dr. M. says, he gives full credit to my own representation, and upon that alone grounds his verdict, I would wish to ask, how he can form such a conclusion, or conceive that a fellow creature could possibly have suffered, when it is asserted that I did not propose to leave my patient till she was so far recovered that my assistance was no longer necessary? Beside which he must have observed, that at the request of the party, however repugnant to my feelings, I did consent to stay, and at the desire of the same gentleman attended twice afterwards. These are strong circumstances against the charge Dr. M. has so uncharitably pronounced; and I have reason to believe, if he has perused the subsequent numbers of the Medical Journal, he will have seen so many proofs of my Opponent's intemperate and contemptuous language, not only towards myself but to Messrs. Wilkinson, Atkinson, &c. that I trust he will be inclined to abate somewhat of that severity which he has manifested towards an unoffending stranger. After all, if Dr. M. doubts my objections to such a consultation to have had any reasonable foundation, and though unacquainted with my character, declares my conduct is not to be justified; I must appeal from such a judgment to the justice of those better able to determine,—to the professional gentlemen in my own county and neighbourhood, and to those who have known me intimately for a long series of years.

I am now to notice some contradictions of Dr. L. upon extravasation or effusion, which he contended was the cause of *that particular case of Apoplexy*, the subject of controversy between us; and which he so *positively* asserted, was invariably present in every attack of real Apoplexy. In *explanation*, however, he says, the two words, extravasation and blood were not made use of by him, because the parties who were present assert they did not hear them; and still more wonderful to relate, because of this circumstance, Dr. L. says, " *the expression being directly repugnant in its import to my opinion.*" I think it wholly unnecessary to trouble the recollection of the parties for evidence which the Doctor himself has afforded me, and which I hope will enable us to establish the truth beyond all argument.

On the 28th of August (two days after the fit) the Doctor says, " We found the patient much better, and without any symptoms of *fresh effusion*, and the pain and oppression of the head even much abated, and I had reason to believe, the principal part of the *effused* fluid was *now* re-absorbed." If the effusion had not at first been asserted to be *considerable*, how are we to reconcile the principal part being absorbed, and *now* but little remaining.

Again " Mr. C. approved of this doctrine, (meaning the leeches) but denied *the extravasation.*"

It is difficult to imagine or to explain how that could be denied, which had not been affirmed.

Again, " I believe I did not say *considerable*, though if I did, the symptoms which Mr. Primrose told me existed *till after she was bled*, were fully equal to support *such an opinion.*"

This surely is not only acknowledging, that he said *extravasation of blood upon the brain in this particular case*, but defending the opinion of its being *considerable*, if Mr. P. was correct; of which there never was any reason to dispute.

I do not mean to animadvert upon Dr. L's physical romance, which he has addressed to Mr. Atkinson, or his strictures upon Mr. Wilkinson; those gentlemen, if they think them worthy of notice, will, I doubt not, correct the Doctor's errors, and fully refute his unfounded assertions.

An admired writer, says, " Surely the greatest arrogance that ever entered into the human heart, is that which not only pretends to be *positive* itself, in points wherein the best and wisest have disagreed; but looks down with all the insolent superiority of contemptuous pity on those, whose *impartial reasonings* have led them into opposite conclusions."

I am, &c.

*Beccles, March 7, 1802.* W. CROWFOOT.

